# Epigenomic insights into common human disease pathology

**DOI:** 10.1007/s00018-024-05206-2

**Published:** 2024-04-11

**Authors:** Christopher G. Bell

**Affiliations:** William Harvey Research Institute, Barts & The London Faculty of Medicine, Queen Mary University of London, Charterhouse Square, London, EC1M 6BQ UK

**Keywords:** DNA methylation, Chromatin, EWAS, Common disease, Ageing, Ageing-related disease

## Abstract

The epigenome—the chemical modifications and chromatin-related packaging of the genome—enables the same genetic template to be activated or repressed in different cellular settings. This multi-layered mechanism facilitates cell-type specific function by setting the local sequence and 3D interactive activity level. Gene transcription is further modulated through the interplay with transcription factors and co-regulators. The human body requires this epigenomic apparatus to be precisely installed throughout development and then adequately maintained during the lifespan. The causal role of the epigenome in human pathology, beyond imprinting disorders and specific tumour suppressor genes, was further brought into the spotlight by large-scale sequencing projects identifying that mutations in epigenomic machinery genes could be critical drivers in both cancer and developmental disorders. Abrogation of this cellular mechanism is providing new molecular insights into pathogenesis. However, deciphering the full breadth and implications of these epigenomic changes remains challenging. Knowledge is accruing regarding disease mechanisms and clinical biomarkers, through pathogenically relevant and surrogate tissue analyses, respectively. Advances include consortia generated cell-type specific reference epigenomes, high-throughput DNA methylome association studies, as well as insights into ageing-related diseases from biological ‘clocks’ constructed by machine learning algorithms. Also, 3rd-generation sequencing is beginning to disentangle the complexity of genetic and DNA modification haplotypes. Cell-free DNA methylation as a cancer biomarker has clear clinical utility and further potential to assess organ damage across many disorders. Finally, molecular understanding of disease aetiology brings with it the opportunity for exact therapeutic alteration of the epigenome through CRISPR-activation or inhibition.

## Introduction

Our ability to associate human genetic variation with common disease susceptibility has been revolutionised since the initial completion of the human genome project. This breakthrough was enabled by the stepwise technological and methodological progress of high throughput Single Nucleotide Polymorphism (SNP) arrays, HapMap informed common Linkage Disequilibrium (LD), and ever-expanding deeply-phenotyped human datasets. This has bought us to the substantial power of the present biobank era (e.g., UK Biobank, FinnGen, NIH *All of US*, etc.) [1]. Today more than ~ 562 k disease associations are listed in the NHGRI-EBI Genome-Wide Association Study (GWAS) Catalog (December 2023) [2].

Despite this success, significant hurdles are still faced in moving from the identified common variant association to a potential pathogenic effector gene [3]. This is principally because ~ 88.5% of NHGRI-EBI GWAS variants are non-coding [4] and are typically in high LD with numerous other co-inherited variants, any of which may be the critical *cis*-regulatory variant(s) influencing a downstream effector target gene(s). The integration of cell or tissue-specific epigenomic data with these initial GWAS findings is a highly informative step in achieving functional understanding [5]. It expedites a narrowing of the focus to those variants residing within disease-relevant tissue regulatory elements [6] and pinpoints the more likely causal genetic variant(s).

This review will illustrate the current successes that have so far arisen though integrative analysis of the epigenome but also the immense potential these data bring for molecular insights in disease mechanisms as well as clinically actionable biomarkers. In summary, this overview firstly describes the functional apparatus of the epigenome. This includes histones, their post-translational modifications as well as amino acid sequence variants, and DNA modifications. It then discusses the integration of these multiple levels of the functional epigenome. The latent regulatory role of transposable elements, comprising half of the human genome, is surveyed, along with the full range of potential genetic variation, and how this can impact on the measured epigenome. Epigenomic association studies with common disease and their complex interpretation are then discussed in detail. The novel epigenomic insights into ageing are considered, including epigenetic analysis of disease-risk through ageing-related changes, as well as quantifying accrued environmental exposures. Finally, the significant therapeutic potential of epigenome-modifying drugs is briefly explored.

## The epigenome is a co-ordinated multi-layered apparatus

### The epigenome is a cell-type specific blueprint

The epigenome comprises the packing and chemical modifications of the genome that influence and inform cell-type specific activity. Whilst all nucleated cells in the human body possess the same genome—except for diversity components of the adaptive immune system and sporadic ageing-related or malignant somatic mutation—the cell-type specific epigenomic blueprint enables or informs on specialised cellular programmes. Thus, each cell, and more broadly organ system, can perform its unique role so that the body can work synergistically together. Epigenetics itself can be defined on a conceptual level as molecular gene activity marks inherited through mitosis or more generally as a systemic persistence of cell-type through genome-wide regulatory networks [7]. An operational understanding of the epigenetic process can be broken down into three components: the action of an ‘epigenator’, or environmental factor; an ‘initiator’ such as a specific transcription factor (TF) and/or non-coding RNA (ncRNA), as a sequence locator; and finally, a ‘maintainer’ such as the epigenetic marks described below, including DNA methylation or specific histone post-translational modifications (PTMs) [8].

### The histone code informs on cell-specific functionality

A major component of the epigenomic apparatus is the histone proteins that the DNA is entwined around—with the unit of a nucleosome containing an octamer of these proteins wrapping ~ 147 base-pairs (bp) of DNA. These form the fundamental components of the complex and dynamic macromolecular structure termed chromatin [9]. The histone proteins (two of H2A, H2B, H3, and H4) also possess protruding tails that are targets for numerous PTMs. Additionally, linker histones (H1) bind the entrancing and exiting DNA from the nucleosome, thereby influencing stability [10]. The tail PTMs can be deciphered as indicators of functional activity, termed the Histone Code [11]. Additions include acetylation (ac), phosphorylation (ph), methylation (me), and ubiquitination (ub) [12]. Further low abundance long-chain acetylation modifications involve crotonylation, lactylation, and succinylation [9]. Modifications of these tails, both causal and consequential, reinforce the local transcriptional state, either through recruiting or repelling specific transcription factors (TFs) or chromatin proteins [9]. Furthermore, by affecting the octamer dynamics, chromatin modifications can influence DNA unwrapping and sliding [13].

**Fig. 1:**
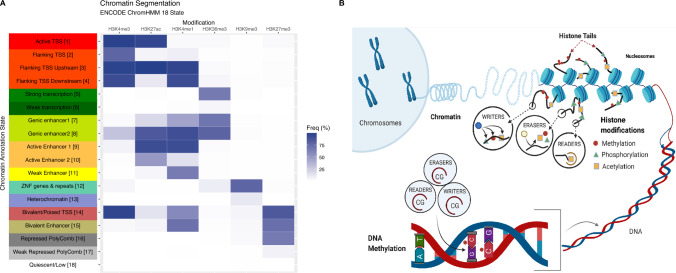
**A** Chromatin Segmentation. Multiple assessed histone modifications are used to predict the chromatin state via ChromHMM (ENCODE 18-state, data from Roadmap Epigenomics, https://egg2.wustl.edu/). **B** The Epigenome. The epigenome consists of histone modifications as well as DNA modifications. Epigenomic readers, writers and erasers are required to precisely co-ordinate the epigenomic machinery (Figure from Berjawi & Bell [55])

The canonical PTMs mostly occur on the tail of the Histone 3 protein at Lysine residues (K) and their identification can infer the location of gene promoters (H3K4me3), enhancers (H3K4me1), and the activation of both these functional units with co-occurring H3K27ac. Transcribed genic regions are marked by H3K36me3. Repressed heterochromatic genomic locations are either indicated by H3K9me3, for permanently condensed constitutive loci found in repetitive, satellite and centromeric regions [14]), or H3K27me3, for facultative reversibly condensed loci [15]). In combination these marks are powerfully informative, enabling machine learning (ML) algorithms (e.g., ChromHMM [16], Segway [17, 18], Epilogos [19]) to predict tissue-specific functional units of the genome. This genomic annotation is termed Chromatin Segmentation (Fig. 1a).

However, these canonical changes are merely the tip of the iceberg, with further complexity entwined in modulations not only occurring in the terminal tails (e.g., in H3, the amino acids numbered 1–44) but also within the globular core (H3 amino acids 45–135) of all the nucleosomal histones [9]. Acetylation within the globular domain is also indicative of activity (e.g., H3K64ac and H3K122ac) [20]. This acetyl modification is observed to destabilise the nucleosome, as H3K64 is positioned on the lateral surface of the histone octamer where the DNA and histone interact strongly [21]. Furthermore, numerous additional novel PTMs have been recently described, including an intriguing potential metabolic role for histone lactylation [22], as well as the brain-related neurotransmitter-based modifications dopaminylation [23] and serotonylation [24]. This discovery of the interplay between these bioactive monoamines and the epigenome brings further fascinating complexity to chromatin-regulatory and neurotransmitter networks [25].

### Histone variants have distinct roles in both physiology and pathology

Multiple copies of each canonical histone gene exist (H2A, H2B, H3, H4 and linker H1), along with genes that have diverged slightly or moderately in amino acid sequence to encode different ‘variants’, as well as non-functional pseudogenes of both canonical and variant versions. Encapsulating all these possibilities, the HUGO Gene Nomenclature Committee (HGNC) database includes a total of 118 in the histone gene group [26]. The canonical genes reside largely in one major cluster (chromosome 6p22), with the remainder found in three minor clusters (1q21, 1q42, 12p12), which also include some of the variant forms. The remainder of the variants are spread across the genome. A critical difference between canonical and variant histones is in expression during the cell cycle, as the former are replication-coupled, or dependent, being synthesised during S-phase, while the latter are replication-independent [27]. Additionally, the canonical forms do not possess intronic sequence or poly (A) tails, in contrast to most of the variant genes [26]. These variants occur in all histone protein families, with, for example, the discovery of the role of the H4.G variant in ribosomal transcription [28]. These substitutions, along with the other epigenomic mechanisms, influence nucleosomal activity [27].

Linker histone H1 bind to specific sites influenced by GC nucleotide content and is the most diverse with 11 variants, with H1.2 favouring exons and high GC whilst, in contrast, H1.3 resides in introns with relatively lower GC [29]. H1 variants modulate RNA Pol II elongation and subsequently affect splicing. In humans, six major variations of Histone 2A exist, with the canonical H2A alongside H2A.X, H2A.Z, macroH2A and two testis-specific variants H2A.B and H2A.P [30]. The well-studied H2A.Z differs in amino acid sequence from the canonical form crucially within the C-terminal tail, increasing DNA accessibility [31]. This variant plays numerous roles, including gene regulation via an influence on initiation and termination, modulating transcriptional efficiency, as well as chromosome segregation and repair processes [32]. Many pathological roles are also observed. These include further subtle H2A.Z subtype substitutions implicated in cranio-facial developmental disorders [33], as well as interplay with PTMs, as H2A.Z acetylation can drive the creation of neo-enhancers, e.g., in prostate cancer [34].

H3 variants have a dynamic role in the cell-cycle [35]. The two major variants are termed H3.1 and H3.3, which display a strong difference between replication timing, with the former enriched at inactive, late-replicating regions and the latter in early replicating sites in active chromatin [36]. This H3.3 variant performs a critical role in development and reproduction [37]. However, as it accumulates in post-mitotic cells, its influence on the histone methylation landscape is implicated in ageing-related alterations of the epigenome. H3.3 is mutated in numerous human diseases, with mouse knockout models demonstrating important roles in chromatin organization and genomic stability [38].

The term ‘oncohistone’ describes the occurrence of frequent somatic mutations in certain histone genes that act as malignant drivers [39]. These have been identified in H3 variants at Lysine 27 or Glycine 34 (e.g., H3.1K27M, H3.2K27M, H3.3K27M, and H3.3G34R/V) in paediatric gliomas, with the differing mutants having specific oncogenic characteristics, regarding their age of onset, anatomical, and clinical features [40]. These mutated histones have high genetic penetrance and are proposed to promote tumour-genesis through their abrogation of key regulatory PTMs, leading to a misregulation of the epigenome and transcriptome [39]. Furthermore, vaccines against oncogenic substitutions are being developed, including a H3K27M-target for diffuse midline glioma in adults [41]. De novo missense variants within histones also are enriched for developmental disorders, with, for example, multiple mutations implicated in H4 genes in neurodevelopmental syndromes [42].

### DNA modifications are robust and stable marks

The direct chemical additions to DNA predominantly occur at cytosine bases in vertebrate genomes [43]. Almost all these modified bases are followed by a guanine in the 5′–3′ direction and thereby assemble as a CpG (cytosine—phosphodiester bond—guanine) base sequence on the same strand. This CpG dinucleotide through its ability to be epigenetically modified functions as a genome-wide signalling module [44]. The major epigenetic change occurring here is the addition of a methyl group to the 5th carbon of the cytosine (5mC) termed DNA methylation (DNAm) [45]. This is through a strong covalent bond and contributes to these marks’ robustness and stability even in long-term sample storage [46].

Additional rarer modifications also exist at CpGs, and these occur through the oxidative action of the TET (ten-eleven translocation methylcytosine dioxygenase) enzyme [47]. It drives the process of active removal of 5mC, initially to hydroxymethylcytosine (5hmC), which may possess a signalling role itself [48], and then on to formyl-(5fC) and subsequently carboxyl-methylcytosine (5caC). This last modification is then removed by the action of the TDG (Thymine-DNA glycosylase) enzyme, returning the cytosine base to the unmodified state [49].

The classical understanding of the addition of methylation to DNA is that the de novo enzymes DNMT3A and DNMT3B enable both strands to be simultaneously modified to 5mC and the maintenance enzyme, DNMT1, through the ability to recognise the single stranded hemimethylated CpGs post replication, recreates the symmetrical methylated palindromic CpG [50]. Lack of DNMT1 action through rounds of replication thereby leads to passive demethylation by dilution. A DNMT2 enzyme is also present in humans, however, its target is exclusively the methylation of transfer RNA (tRNA) [50]. The gene bodies of expressed genes possess significant DNAm. DNMT3B facilitates this targeting of transcribed regions of the genome via its PWWP domain, which interacts with SETD2-mediated H3K36me3 also co-localised to these genic regions [51]. This interplay illustrates the co-ordinated nature of both the chromatin and DNA based epigenome [52]. An additional enzyme DNMT3L, a truncated non-active methyltransferase, due to the lack of the functional catalytic domain, acts as an activity cofactor for DNMT3A, forming a heterotetrameric complex [50]. The distinct maintenance and de novo roles of DNMT types 1 and 3 do, however, erode to a certain extent in vivo with the observation that localised DNMT3 is also required for the maintenance of DNAm at imprinted and repetitive loci [53].

Although rare, non-CpG cytosine methylation also occurs, observed in developmental, germ cells, as well as neuronal tissues, where the scale of epigenomic complexity is still undefined [54]. The level of non-CpG methylation is correlated with the level of expression of de novo DNMT3 enzymes. Whilst these epigenomic writers possess a strong template preference for CpG, with persistent action they will then subsequently methylate CpAs followed by CpTs, as observed in non-dividing neurons [56]. This signature in non-replicating cells is consistent with a lack of a known maintenance mechanism through replication for non-CpG methylation, as the structure of DNMT1 enforces a strong preference for hemimethylated CpG dinucleotide action only [57].

### Mutation of epigenomic machinery genes leads to significant pathology

Alongside the DNMT and TET enzymes, described above, vital in constructing the observed DNA methylome, numerous core families of PTM enzyme writers (e.g., lysine methyltransferases, KMTs; lysine acetyltransferases, KATs) and erasers (e.g., lysine demethylases, KDMs; histone deacetylases, HDACs) also exist. Therefore, these writers, and erasers, along with additional readers and remodellers, are critical to the operation of the cellular epigenomic apparatus (Fig. 1b). Mutation of these critical genes leads to monogenic disorders, termed the Mendelian Disorders of the Epigenetic Machinery (MDEM) [58]. MDEMs are enriched for developmental disorders [59], particularly neurological phenotypes, but are also observed as well as oncogenic drivers [60]. For instance, a high mutation rate was recently identified in the mammalian SWI/SNF ATP-dependent chromatin remodelling family of genes in neurodevelopmental disorders and these cluster within key structural domains [61]. Further exploration of the abrogation of these critical MDEM genes has brought novel molecular insights into specific epigenetic modifications, including observed phenotype convergence [62]. Ageing-related somatic mutations in the epigenetic DNA methylome machinery, specifically *DNMT3A* and *TET2*, are observed to be strong drivers of ageing-related clonal haematopoiesis [63]. These clonal expansions of mutated blood cell lineages are a risk factor for malignant transformation over several years, with a ~ 10-fold relative-risk increase for myelodysplastic syndrome, acute myeloid leukaemia, myeloproliferative neoplasms, and certain lymphomas.

### Functional tissue and cell-type regulomes have been derived from the construction of epigenomic reference maps

Second generation Illumina short-read sequencing (2nd Gen-seq) accelerated the epigenomic era by enabling the whole genome base pair resolution assessment of histone PTMs, DNAm, transcription factor (TF) binding, and chromatin accessibility [64]. The first DNA methylome, i.e., DNAm at genome-wide level, using whole genome bisulphite sequencing (WGBS) was published in 2009 [65]. The surveying of the canonical epigenomic state of specific cell-types has progressed significantly through multinational consortium endeavours—beginning with the ENCODE project [66] in cell line data, and then the Roadmap Epigenomics [67] in tissue samples, as well as more specialised accomplishments in the haematopoietic system via the Blueprint Consortium [68] and the brain via PsychEncode [69]. The International Human Epigenome Consortium (IHEC) portal provides access for  ~ 7.5 k hg38 publicly available datasets (https://epigenomesportal.ca/ihec/) [70].

### The haploid DNA methylome is now estimated to comprise ~ 33.9 million CpGs

Long read single molecule or third generation sequencing technology, (e.g., Oxford Nanopore, ONT; and PacBio HiFi; 3rd Gen-seq) can assess both sequence and DNA modification directly. A recent telomere-to-telomere genome assembly (T2T-CH13 cell-line) derived from a duplicated haploid hydatidiform mole with a 46, XX karyotype has allowed analysis of a further ~ 8% of the human genome [71]. With the ability to traverse of centromeric monomers and other repetitive elements, and with the recent T2T addition of chromosome Y, this has brought the haploid estimate of CpGs up from ~ 28–30 million to ~ 33.9 million (T2T-CHM13v2.0) [72–74]. Additionally, realigning to this T2T assembly also increased the number of chromatin ChIP-seq peaks from ENCODE, mostly H3K9me3, located in peri/centromeric satellite constitutive heterochromatin, but to a lesser extent also facultative heterochromatic H3K27me3 loci.

### DNAm variability is tissue-specific and is not focused in CpG dense ‘CpG islands’

The advent of large scale epigenomic datasets has bought new insights beyond the single gene cancer and imprinted loci research that was the focus of previous decades. These prior targeted studies, due to the methodologies available, had primarily analysed the CpG dense regions, termed ‘CpG islands’ (CGI), observed to occur at ~ 70% of vertebrate promoters [75]. CGIs are ~ 1–2 kb in size [76] and possess a CpG density as percentage of sequence ranging from ~ 12% up to over 30% (i.e., 100% = 50 CpGs dinucleotides per 100 bp) [77]. These clustered ‘island’ outliers only comprise ~ 7% of the total set of CpGs (UCSC CGI definition) [78]. They protrude sporadically amidst the mostly sparsely spread ‘open sea’ CpGs. CGIs act as a platform for transcription [79], facilitating this process, although the majority of CGI are unmethylated independent of their transcriptional activity [80]. However, within CGI themselves there is a further correlation in DNAm level, with higher CpG density CGI promoters remaining consistently unmethylated and comparatively lower CGI promoters being those that are more likely to be targeted to gain methylation [75], such that these ‘weaker’ CGI are predisposed to acquire DNAm during differentiation [80].

The baseline template for the state of the DNA methylome is therefore CpG density, with most CpGs at low density and methylated, whilst conversely the clustered ‘islands’ remain unmethylated [81]. Longstanding estimates have stated ~ 70–80% of all CpGs in the genome are methylated [82]. Analysis of 42 Roadmap WGBS across 32 cell and tissue-types supported this, identifying that the vast majority of CpGs were static and highly methylated, with only ~ 21.8% CpGs being ‘dynamic’ with DNAm changes of ≥ 30% in autosomes [83]. A further evaluation in the human body DNA methylome analysis of 36 WGBS DNA methylomes across 18 tissue-types from 4 individuals arrived at a similar estimate of ~ 15.4% of CpGs changing by ≥ 30% between tissues [84].

**Fig. 2:**
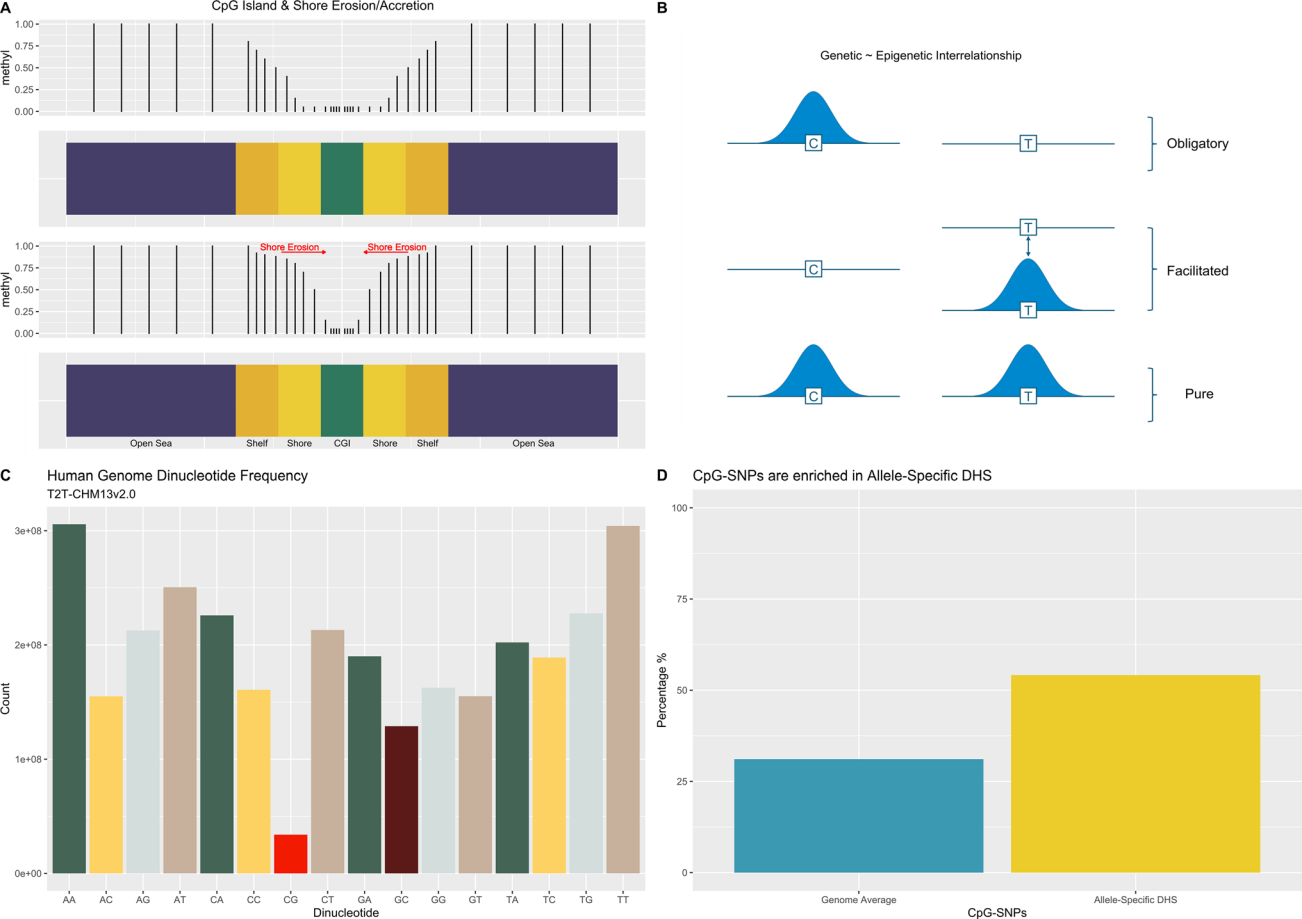
**A** CpG Island Shore Erosion and Accretion. DNA methylation gain at CpG islands commonly occurs from the outer shores inwards, eroding the shore’s relative hypomethylation state. The inverse occurs when DNA methylation is lost, leading to shore accretion as the hypomethylation spreads out. **B** Genetic—Epigenetic Interrelationship. Obligatory epigenetic states can be directly predicted by genotype (e.g., C & T examples here). Facilitated states enable epigenetic variation associated with one genotype, but not the other. Pure epigenetic variation is under no genetic control (Adapted from Richards [155]). **C** Human Genome Dinucleotide Frequencies (T2T-CHM13v2.0). A significant depletion in CpG dinucleotides (in red) is observed due to their DNA methylation-associated hypermutability. **D**. CpG-SNPs. Compared with their genome average (~ 31.1%), CpG-SNPs are enriched to reside within TF motifs that modify TF binding and lead to allele-specific DNase-I Hypersensitivity Sites (DHSs) (~ 54.1%) [167]

With CGIs themselves being consistently unmethylated across all tissues, except for specific outliers, it is the regions that surround them, termed CGI ‘shores’ (± 2 kb), where significant DNAm variability is observed, and this also correlates with gene expression [83, 85, 86]. These surrounding regions are the CpG density transition zones between islands (> 12% CpG density) out to the genome average ‘open sea’ regions (~ 2%). Additionally, CGI ‘shelves’ are delimited as the regions just beyond shores, 2–4 kb either side of the islands. Distinctly visible in WGBS data is the tidal nature of DNAm in cellular differentiation. Hypermethylation progresses inwards reducing the width of the unmethylated proportion of these shores and then island edges, with outgoing hypomethylation having the opposite effect [87] (Fig. 2a). Though, a further subtlety beyond simple designated CGI shore regions was proposed by the human body DNA methylomes analysis, with the strongest negative correlation with gene expression localised to be specifically in the downstream mid-shore region ~ 0.3 kb and spreading as far as 8 kb intragenically [84]. Induction of DNAm via a Zinc Finger (ZF) with a tethered methylating DNMT3A enzyme in an experimental model has further illustrated the complexity of the DNAm-transcription relationship with highly context specific promoter effects [88]. Classic promoter DNAm-coupled repression was observed, as well as supporting previous data that ejection of methylation-sensitive TFs, such as NRF1 [89] contributes to repression. However, this study also detected DNAm leading to the upregulation of genes enriched for methylation-sensitive transcriptional repressors in their promoters. Recent synthetic models modulating CpG density and methylation state support a strong correlation between silencing and high CpG content, but also indicate potential sequence resolution intricacies with certain CpGs disproportionally controlling silencing [90]. It has also been recognised that CGI promoters can influence transcription through a CGCG tetranucleotide motif associated with highly expressed genes that binds the BANP (BTG3-associated nuclear protein) TF [91]. A further novel role of CGIs was recently discovered, where binding by the protein complex SET1 protects genic transcripts from premature termination [92].

### Observed co-ordination between CpG island (CGIs) sequence and chromatin state

The multiple layers of the epigenome and DNA sequence are an intricately interconnected and co-ordinated system [52]. Illustrating this, chromatin modifying factors containing a ZF-CxxC domain, such as CXXC1 (Cfp1), an essential component of the SET1A/B H3K4 histone methyltransferase complex, are recruited to unmethylated CpG motif clusters [93]. This recruitment results in the trimethylated chromatin signature, H3K4me3, which is strongly associated with CGI gene promoters and aids transcription [92] as well as Pol II pause release [94]. This role of clustered unmethylated CpGs attracting CXXC1 means dramatic human-specific increases in CpG density can predict loci with human-specific H3K4me3 peaks [95]. Furthermore, the CpG density of a CGI promoter is indicative of its functional importance, with higher density CGIs significantly more likely to be serving loss-of-function (LoF) intolerant genes [96].

### Distal cis-regulatory elements (CREs) can be detected by reduced DNAm levels

Distal *cis*-regulatory elements (CREs) can be detected with WGBS data through discrete and more subtle reductions in DNAm level. Low methylation regions (LMRs) averaging ~ 30% DNAm and possessing above genome baseline CpG densities of ~ 2.5–5%, are enriched for the enhancer locating H3K4me1 mark, as well as the active chromatin signature of H3K27ac [97]. These loci also display increased levels of the enhancer-associated active protein p300, as well as TET1 and its product 5hmC [97]. The binding of cell-type specific TFs to these regions can lead to a loss of DNAm. These regions have also been termed hypomethylated regions (HMRs) [87], alongside concomitant cell-type specific H3K27ac enhancer signatures [98]. The loss of DNAm is proposed to occur after nucleosome repositioning [98]. In terms of distal CREs or enhancers, the regulatory role of DNAm is proposed to be highly context dependent [99]. Deciphering this *cis*-regulatory code is now being aided by ML and large-scale functional assays [100].

### DNAm directly influences transcription factor binding

Recent evidence indicates that the major repressive mechanism on DNAm-sensitive TFs is principally through the direct inhibition of methylation-sensitive TFs by DNAm, rather than indirectly through the action of methyl-CpG-binding domain (MBD) proteins [101]. However, other TFs may instead be influenced by chromatin state. The TF and tumour suppressor p53 (TP53), a pioneering factor, is not inhibited by DNAm, but instead is restricted by its co-factor Trim24, which itself binds to unmethylated H3K4 [102]. Therefore, the methylation of H3K4, found in accessible chromatin, disables this chromatin-sensitive cofactor and subsequently the p53 TF from occupying this locus. Experiments in mouse stem cells support the premise that both levels of epigenomic activity are important, in that the inhibition of histone deacetylation complexes (HDACs) as well as the inhibition of DNAm led to an additive widespread increase in active chromatin [103].

### The DNAm influence in transcription factor bindings sites is not always repressive

Whilst the classical interpretation is that DNAm is deemed to be repressive for TF binding within CREs, this picture is considerably more complex. As indicated, a portion of mammalian TFs are insensitive to repressive epigenetic marks and can bind inaccessible chromatin enabling subsequent downstream activity [104]. Furthermore, a systemic analysis of the direct relationship between DNAm and 542 specific human TFs, identified that ~ 60% bound to one or more TFBS where CpG methylation was influential [105]. Approximately 26.8% of TFs possess at least one CpG with their binding motif [106]. Whilst some classes of TF were inhibited by DNAm, others, especially those with critical roles in embryonic and organismal development, showed a preference for binding to templates containing methylated CpGs. This latter grouping included the Yamanaka pluripotency factor Oct4, binding a mCpG TFBS motif [107] and, additionally it has been proposed to exhibit a preference for 5hmC [108]. Although Oct4 is not exclusive to this motif, as it recognises other TFBSs that do not contain a CpG [106].

In-*vivo* analysis identified NRF1 to be a highly DNAm sensitive TF [89]. Furthermore, TF sensitivity to DNAm can be positionally dependent on the specific location of the mCpGs within the TFBS motifs [106]. Alongside this is an emerging model where certain TFs bind to mCpG containing motifs and induce demethylation through the action of TET [106].

### Partially methylated domains (PMDs) contribute to the observed global hypomethylation in the cancer DNA methylome

The higher resolution data possible with WGBS DNA methylomes has also revealed larger scale reductions in DNAm in specific biological context termed Partially Methylated Domains (PMDs). These include the placenta [109], cancer cells [110] and cell line culture [111]. Individually they have a mean size ~ 150 kb but can comprise sizeable portions (20–40%) of the genome [112]. They occur in lamina-associated, late-replicating regions. The DNAm loss is contributed to by reduced propensity to maintain DNAm at sparse CpGs, particularly within a specific nucleotide context, termed ‘solo-WCGW’, where W represents weak A or T flanking bases [113]. This loss of methylation in these late replicating regions tracks mitotic turnover and is termed a ‘hypoClock’ [114]. Of note, critical ‘escapee’ genes are observed that can exist within these regions but avoid their neighbouring epigenetic effects [115]. Furthermore, these PMDs are observed to regain DNAm when reprogrammed to a pluripotent state [116].

## Integration of epigenomic data with GWAS findings

### Chromatin segmentation identifies pathologically relevant Cis regulatory elements (CREs)

Epigenomic analysis, including Chromatin Segmentation, enables the identification of cell-type specific CREs, including promoters, enhancers, and insulators. CREs comprise the binding sites for regulatory factors, such as TFs, that initiate and maintain transcription [117]. Deciphering the gene regulatory code that enables CRE activity involves examining how TFs bind to Transcription Factor Binding Site (TFBS) motifs with variable efficiently, co-operating with mediating co-factors, as well as the interplay with chromatin state and nuclear organisation [118]. The construction of a cell-type specific ‘regulome’ though chromatin segmentation by the combined analysis of the epigenome (chromatin PTMs, DNAm, ATAC, DNase-I hypersensitivity sites/DHS) [119] enables integration and interpretation of GWAS signals in the pathogenically relevant tissue. For example, the formulation of a pancreatic beta cell regulome is highly relevant for Type 2 Diabetes (T2D) GWAS evaluation [120].

### Epigenomic integration and interpretation of GWAS signals

Non-coding variants contribute most of the common genetic susceptibility, for example in cardiovascular disease, this is estimated to be ~ 90% [121]. Causal GWAS variants are proposed to subtly modulate regulation of CREs. However, the identification of the target effector gene(s) of a disease implicated CRE is a difficult process and numerous evolving methods have been implemented and continue to evolve including EpiMap [19], ABC [122], and the ENCODE-rE2G methodology [123], etc. Data from the pathogenicity relevant tissue is paramount in this process. Chromatin conformation experiments have been employed, such as promoter-capture Hi-C, whereby potential distal CRE loci that interact with specific protein coding gene promoters are identified [124]. Recently, the combination of statistical fine-mapping and Roadmap-derived chromatin segmentation as well as promoter-capture Hi-C across the range of blood pressure-related tissues enabled the identification of numerous novel effector genes for this common disease [125].

The EpiMap analysis in the Roadmap consortium dataset across 33 tissue categories identified candidate CRE loci where the active H3K27ac mark was present and this quantitatively correlated with a target gene’s expression within 1 Mb [19]. From the 18-state chromatin segmentation, the five enhancer categories (G1, G2, A1, A2, and Wk) were intersected with DHS data to define high-resolution active-enhancer regions. Active enhancer marks that correlate with gene activity levels defined tissue-specific enhancer-gene modules and were then employed to identify potential GWAS effector target genes. The Activity-By-Contact (ABC) model prediction is calculated on the strength of enhancer activity and the frequency of the contact with gene promoter considering all the elements within 5 Mb of the gene [122]. This is informed by contact data, in this case via CRISPRi-FlowFISH that perturbs and assesses enhancers through CRISPR inhibition (CRISPRi), RNA fluorescence in situ hybridization and flow cytometry [122].

The gLink scores methodology was constructed starting with GTEx tissue data profiled across three tissue types (heart, muscle, lung) for the active chromatin mark H3K27ac to define Active Regulatory Elements (AREs) [126]. Genetically influenced AREs were identified indicating ~ 130 k haQTLs (histone acetylate QTLs). Identified GWAS-haQTL-colocalised gAREs and ARE-gene linking scores were constructed via multiple strategies, including impact on expression in the same tissue as well as proximity to an eQTL, shared genetic regulation between expression and AREs, and also utilising previous EpiMap or ABC scores. This prioritisation relies on interindividual genetic variation, not the inter-tissue activity variation as with EpiMap. Of note is that in identifying cell-specific function, bulk haQTLs were found to have advantages over bulk eQTLs. This was proposed to be due to the expression effect potentially only manifesting under disease conditions and/or cell types, as opposed to the potentially persistent permissive regulatory signal [126].

A heritability informed investigation of available GWAS SNP-to-Gene (S2G) methods including seven previous S2G approaches was constructed as a combined S2G (cS2G) linking strategy [127]. Through linear combinations of individual linking scores, this concluded that the combined framework could double the success of any individual methodology. Subsequently, to foster further understanding of the human enhancer to promoter relationship, sequence compatibility of CREs was assessed through enhancer by promoter self-transcribing active regulatory region sequencing (ExP STARR-seq) [128]. This concluded that in contrast to housekeeping genes, whose promoters were less reactive, variability expressed genes responded strongly to enhancers. The enhancers themselves had similar activity on promoters but could combine multiplicatively thereby increasing transcription. The Polygenic Priority Score (PoPS) to prioritise protein-coding effector genes was also recently proposed [129]. It is informed by cell-type-specific expression data along with biological pathways and protein–protein interactions. This method was successfully used to classify gene–trait pairs across > 100 complex traits and successfully recapitulated known findings.

The most recent prediction is the ENCODE-rE2G methodology [123]. This was constructed by using ENCODE data comprising > 13 million enhancer-promoter interactions from 352 cell types and tissues, chromatin segmentation and 3D contact data as well as CRISPR perturbation experiments, fine-mapped eQTLs and GWAS SNP data. This analysis has provided a human genome encyclopaedia of enhancer-gene regulatory interactions. Additionally, characteristics found to be important, were again promoter class (ubiquitous housekeeping or variably expressed) as well as enhancer-enhancer synergy. Moving forward, computational methods, as opposed to experimental data, will be increasingly used to predict effectors, such as neural network Deep Learning (DL) for de novo prediction of cell-type-specific chromatin organization [130], or DL for TF binding prediction [131].

### Single-cell analysis is increasingly used to identify cell-specific CREs

CREs categorisation is also gaining traction through single-cell (sc) analysis [132]. This includes scDNAm via droplet-based bisulphite (BS)-converted DNA [133] (microfluidic Drop-BS) [133]. For example, a scDNAm human brain analysis [134] utilised methylation barcodes (scMCodes) to identify human brain cell types alongside sc-ATAC-seq for cell type specific functional interpretation [135]. Additional advances include the analysis of non-coding variants at scale by single-cell CRISPR screens [136].

## The epigenome of the repetitive human genome

### Transposable elements (TEs) can modulate the state of the surrounding epigenome as well as function as CREs

Transposable elements (TEs) are estimated from the recent T2T-CH13 assembly to comprise 53.9% of the human genome [71]. DNAm is a fundamental mechanism in repressing the aberrant function of these sequences [137]. Furthermore, these repeats can disseminate their epigenomic state to the surrounding genomic region, with evidence for modulation of nearby gene transcription from various TE classes, including *Alu* elements [138], SVAs [139], and LINE1s (L1s) [140], as detailed below.

Inserted TEs can be co-opted into functional CREs [117]. They can act as promoters, enhancers, and silencers, as well influencing 3D genomic architecture through, for example, short-interspersed element (SINE)-derived CTCF sites [141]. TEs that reside intronically are shown to be enriched as tissue-specific enhancers [142].

Approximately a quarter of all CpGs (~ 7.5 M) reside within the ~ 1.2 M *Alu* elements, a ~ 300 bp sized primate-specific SINE [143]. *Alu*s comprise ~ 10.1% of the human genome (T2T) [144]. Numerous TFBSs reside within its sequence context [145] and this element is observed to have enhancer potential [146], with epigenetic de-repression influencing nearby gene expression [138, 147]. De novo insertion of *Alu* elements is significant in several developmental disorders [143]. Additionally, population polymorphic *Alus* are enriched within GWAS loci, with examples identified that are in strong LD with the GWAS led SNP [148]. *Alu* elements are predominantly methylated across all normal tissues, with estimates of ~ 1–4% unmethylated *Alus*, (1.0–1.6% in the blood), and only ~ 0.3% consistently unmethylated across all samples [149]. In cancer cells they are more resistant to hypomethylation, compared to other repetitive elements, implying strong pressure to maintain their epigenetic state [149]. When loss of DNA methylation does occur, it is preferentially from its 5′ and 3′ ends [150].

SVAs are a composite ~ 2 kb element containing a SINE, a variable number tandem repeat (VNTR), and an *Alu* element [151]. They arose ~ 25 million years ago (MYA), are hominid-specific (i.e., the existent and extinct great apes), and although only comprising ~ 0.15% of the human genome (T2T) [144], with ~ 3700 copies [139], have had a disproportionate influence in human genome evolution [152]. They are frequently co-opted into regulatory roles in the human genome [139]. In a great ape comparison, humans show a strong enrichment for CpGs that are human-specific within SVAs [153].

LINE1s (L1s) are larger ~ 6 kb, accounting for ~ 16.8% of the human genome (T2T), and by contrast with *Alus* and SVAs are autonomous in their ability to transcribe themselves, although if they become inactive through 5′ truncation their CpG content reduces rapidly over time [144]. L1s are associated with tissue-specific regulatory elements [154]. Increased gene body methylation, through DNAm of intronic L1s is correlated with transcriptionally active genes. As well L1 insertions have been observed to spread DNAm to proximal upstream regions [140].

## Genetic influence on the epigenome

### Genetic variation in epigenome can drive or enable epigenetic function

Genetic influence on the epigenome can be defined into three categories, pure, facilitated, and obligatory (Fig. 2b) [155], This concept needs careful consideration with respect to all the methodologies used to assess the epigenome. ‘Pure’ epigenomic variation is under no genetic influence and could represent the variation between an active and dormant enhancer with the former possessing significant H3K27 acetylation from the latter. A genetically ‘facilitated’ enhancer could be present due to sequence variation that constructs a motif for an enhancer-activating TF on one allele that is not present on the converse allele. An ‘obligatory’ enhancer could be present due to a portion of active regulatory sequence that is present on one allelic background and completely deleted on another. Therefore, all the quantitative sequencing approaches that depended on fragment enrichment, such as ChIP-seq, MeDIP-seq, etc., will strongly detect obligatory epigenetic states in any differential analysis.

Both obligatory and facilitated genetically epigenetic changes can be misinterpreted as pure epigenetic changes. This can be starkly illustrated by the early observations that proposed that imprinting abnormalities were a common occurrence in cancer genomes, such as the loss of *IGF2* imprinting as a potential biomarker of colorectal cancer risk [156]. However, more nuanced recent analysis has identified that the abnormal methylation profiles at these imprinted differentially DNA methylated regions (DMRs) were not pure epigenetic changes but driven by underlying copy-number aberrations [157].

### Genomic sequence constructs the DNA methylome template and contributes to allele-specific variation

The interplay between genome and epigenome is complex process that is location, time, and cell-type dependent. Allele-specific expression (ASE) is influenced by both genomic and/or epigenomic variation [158]. The widespread influence of allele-specific DNAm (ASM) was first documented in 2010 [159], with the strong contribution of CpG-SNPs to this phenomenon also acknowledged at this time [160], as well as the first recognition of DNAm differences between risk and non-risk haplotypes within a GWAS loci [161]. Allelic variation in the DNA methylome and chromatin PTM landscape has been explored in the reference epigenomic maps [162]. Whilst, as detailed, CpG density indicates broadly the baseline state of the DNA methylome, and CpG density of a locus is fundamental in the interpretation of the potential role of DNAm [163], additional sequence differences can contribute to the next level of epigenetic variation. Within CpG dense regions, methylation determining regions (MDRs) indicate specific TFBSs, such as SP1, CTCF, and members of the RFX family, that when mutated lead to an increase in DNAm [164]. Further allelic effects through the modulation in the gradient of change in CpG density will influence the local DNAm state. Inserted CpGs influence the regional DNAm state, as can be observed through the impact of a CpG dense L1 element acting as a hypomethylated CGI. This insertion will lead to a gradually reduced hypomethylation in the surrounding ‘sloping shore’ region [165]. These density effects are also apparent between neighbouring CGI within 3 kb of each other. The influence of CpG dense polymorphic TEs therefore can contribute to considerable allelic variation in DNAm.

Short Tandem Repeats (STRs, or microsatellites) are seen to influence the local DNAm state, with the classic pathological example of the hypermethylated expanded CGG trinucleotide repeat in the 5′UTR of *FMR1* repressing transcription in Fragile X syndrome (FXS, MIM: 300624). Approximately ~ 11.8 k STRs affecting the local DNAm state have been observed with ~ 12% of these influencing nearby gene expression [166]. Significant variation in DNAm between GWAS risk and non-risk disease-associated haplotypes is implicated to be driven by population variation in STRs [167]. STRs can also act as ‘rheostats’, particularly if they include methylatable CpGs, and tune the local TF concentration [168]. This genetically driven trait-associated DNAm variation can also be due to polymorphic CNVs, Indels, as well as clustered in-phase CpG-SNPs [167]. These variants can also impact on chromatin enrichment analyses, such as ChIP-seq as well. Structural variants can influence the promoter-enhancer connections by modulating 3D interactions [169]. Extensive differences in chromatin conformation between heterozygous loci were recently recognised with allele-specific topologically associated domains (TADs) contributing to allele-specific expression [170]. Population level integration of regulatory and sequence data is currently lacking, as even small scale haplotypic analyses of multi-tissue personal epigenomes indicate (e.g., EN-TEx resource in four individuals) [171]. Larger scale single tissue analysis in peripheral blood has further indicated this population variability in the epigenome, e.g. analysis in > 3000 DNA methylomes via the antibody enrichment based MeDIP-seq method identified 7173 Haplotype Specific DNAm (HSM) peaks that differed between GWAS disease or trait risk and non-risk haplotypes [167]. Of these peaks, ~ 37% overlapped non-SNP variants (CNVs, STRs, Indels) and comparison across chromatin segmentation for six core ENCODE tissues revealed ~ 10% coincided with enhancer signal. A recent analysis of population variability of VNTRs in regard to phenotypes identified 4968 significant VNTRs [172] and 143 of these intersect with HSM peaks.

### CpG dinucleotides are depleted genome-wide and enriched as CpG-SNPs

The CpG nucleotide, the major template for vertebrate DNAm, is depleted to ~ one quarter of its expected frequency [64]. This is due to the hypermutability of methylated cytosines, as the spontaneous deamination of 5mC occurs at ~ 14-fold greater rate than other single nucleotide substitutions in humans [173]. This mutational strength is starkly apparent when compared to the inverse dinucleotide GpC, which not being a direct target for methylation occurs almost four times more frequently than CpGs (T2T CHM13v2.0: CpGs ~ 33.9 M, GpCs ~ 128.9 M, Fig. 2c). Thus, the methylation state of the germline cells (sperm [174] and ovum [175]) is a critical factor in this mutational force. Approximately 30% of all common SNPs reside within a CpG [167], deaminating to YpG (Y = Pyrimidine: C or T) or CpR (R = Purine: G or A) dinucleotides. This mutational change is also observed to create TFBSs at a high frequency, including those for key TFs such as Oct4, NANOG, and c-Myc [176]. However, as high CpG dense promoter regions also remain largely unmethylated in germline tissues, they consequently mutate at a lower rate [75].

Analysis of active open chromatin across multiple tissues via DHSs has revealed that TFs occupy regions of DNA that are hypermutable and that CpG dinucleotides are strong drivers of this focused genetic diversity [177]. Furthermore, consistent with this, CpG-SNPs are  enriched to be SNPs that modify TF binding and lead to allele-specific DHS [167] (54.1% *cf*. their genome average of 31.1%, Fig. 2d). This mutational tendency within activity regulatory loci, seen across mammals, supports the hypothesis that the genome is primed for regulatory evolution [177].

### Solving the ‘Missing Regulation’ of GWAS signals will require population scale epigenomic maps

Expression Quantitative Trait Loci (eQTLs), where gene expression is associated with a SNP’s genotype status, offer a straightforward interpretation of gene-regulatory mechanisms in non-coding GWAS findings. Their potential is implied when the tagging SNP and eQTL are in high LD with each other. However, the utility of eQTLs has undergone a substantial recent reappraisal. This is due to the observation, with now the wealth of data from large-scale GWAS and resources, such as GTEx, that their pathogenic attribution for GWAS signals is only relatively small [178]. An interpretation of this lack of pathogenicity, informed by evolutionary biology, is that common genetic variants correlated with a large change in expression are likely only to be observed in non-critical genes [178]. More broadly, a substantial portion of ‘missing regulation’ is now documented in GWAS interpretative studies, due to unresolved mechanisms [179]. This has also been attributed to context dependence, non-linear and non-homeostatic gene-expression relationships, as well as ancestry related variation [180]. Moreover, a further under-estimated factor is the impact of population obligatory and facilitative genetic variation on the regulatory epigenome due to inadequate population diversity in currently available datasets [181]. The current epigenomic reference maps are derived from sub-optimal cell-line data or very small numbers of tissue samples, especially in comparison with the contemporary power of genetic datasets.

### Array analysis confirms that the genetic influence on the DNA methylome is extensive

DNAm QTLs (mQTLs) are loci where variation in genetic sequence is correlated with variation of specific cytosine DNAm levels. These are predominantly proximal, *cis* acting, but can also be distal or even act in *trans*. These mQTL, as well as haplotype-dependent allele-specific DNA methylation (hap-ASM; also termed HSM) are used in GWAS integrative interpretative analyses [182]. These studies through the overlap of DNAm variation and CTCF insulator loci have highlighted the role of allele-specific CTCF in potential population-specific 3D interaction [167, 182].

In terms of mQTLs themselves, an early integrative analysis explored ~ 3800 individuals from a Dutch Biobank with genotype (~ 5.2 M SNPs), RNA-seq and DNAm 450 k array data [183]. This study identified that of the tested ~ 405 k CpGs, ~ 34% were under the influence of *cis*-mQTLs (~ 140 k CpGs). The median distance for this *cis*-acting SNP from the CpG was ~ 10 kb but peaked strongly around the site of the CpG itself. Of these mQTLs, 1.6 and 0.9% were mixed *cis*- and *trans*- or only *trans*-mQTLs, respectively. For each genetically influenced CpG, up to 16 independent *cis*-mQTL SNPs could identified. Approximately 12 k CpGs correlated with the expression of ~ 3800 genes in *cis* (expression quantitative trait DNAm loci: eQTMs) and this was mostly, but not exclusively, a negative DNAm correlation with expression (~ 70%). Investigating ~ 1900 GWAS disease or trait-associated SNPs determined these affect the DNAm of ~ 10 k CpG sites *in trans*. Variants were identified that were both *cis*-eQTLs for TFs as well as being *trans*-mQTLs for CpGs within the bindings site of these TFs, including CTCF, NFKB1, and NKX2-3.

This substantial influence of genetics on DNAm was further reinforced with another analysis in blood (n =  ~ 4700), which identified ~ 4.7 M non-independent SNPs from a genetic array of ~ 8.5 M SNPs as being *cis*-mQTLs for ~ 120 k from ~ 415 k CpGs (~ 29%) [184].

A meta-analysis for mQTLs in blood derived DNAm with the 450 k DNAm array, incorporating 36 studies, including the above Dutch cohort, was performed in 2020 [185]. This consolidated on ~ 420 k CpGs analysed in ~ 32.8 k samples and using LD clumping identified > 270 k independent mQTLs with now ~ 45% of the array probes identified as being under genetic influence (~ 190 k CpGs). Whilst ~ 248 k independent *cis*-mQTL were identified, the increase in power was even more stark for the *trans* analysis, with now 23 k *trans*-mQTLs (~ 8.5%). Strict statistical thresholds were required in these analyses, i.e.,* p* < 1 × 10^–8^ and 1 × 10^–14^ for *cis*- and *trans*-mQTL analysis, respectively. Therefore, it is highly probable that with further increases in power, these DNAm array by genotype studies will identify the vast majority of the assayed CpGs to be under some level of genetic influence.

A multiple tissue analysis was performed in the Genotype-Tissue Expression (GTEx) dataset, benefiting from analysis across 9 tissues, with ~ 1 k samples analysed in 424 human subjects. Although there are power constraint considerations, due to both smaller and variable numbers across the samples, this analysis identified ~ 286 k CpG mQTLs for > 750 k CpGs from the EPIC v1 (850 k) DNAm array, but with only ~ 5% indicating some tissue specificity [186]. An African American analysis also further supported the substantial genetic influence with ~ 4.5 M non-independent *cis*-acting mQTLs on ~ 321 k CpGs from the EPIC v1 DNAm array identified, with these mQTLs also peaking sharply around the CpG site [187].

A smaller study but in isolated primary monocytes directly explored population differences in DNAm between European and African individuals (n = 156) [188]. Most of these ancestry-related differences (~ 70%) were identified to be driven by nearby differences in sequence variation (i.e.,* cis*-mQTLs). Furthermore, a role for DNAm in immune response regulation was observed with immune stimulation leading to epigenetic changes that associated with the expression of 230 genes. These eQTMs were themselves strongly enriched for genetic control (OR ~ 33.2). Additionally, the effect of the *trans*-master regulator CTCF was observed through SNP rs7203742 to influence the DNAm of 30 distant CpGs.

### The influence of population genetic variation on the DNA methylome is still underestimated, but all cannot be a priori assumed functional

A recent study jointly analysed seven multi-omic layers via blood-related QTLs, e.g., eQTL, mQTL, caQTL (chromatin accessibility, i.e., ATAC), hQTL (histone PTMs), sQTL (splicing), apaQTL (alternative polyadenylation), and pQTL (protein) with summary GWAS data for 50 traits (n = ~21–766 k) [189]. DNAm associations were observed to be comparatively more frequent than other layers. Using windows of 2 Mb centred on the trait-associated independent SNPs and the 7 layers of multi-omic data (n = 0.1–32 k), ~ 50% of these GWAS signals shared at least one QTL. This comprised of ~ 39% that had a DNAm association, twice that of eQTLs at ~ 20% and only 3.4% for pQTL. Furthermore, this was in a restricted array dataset of only ~ 91 k CpGs for DNAm. Loci with multiple shared QTLs have stronger regulatory potential and a priori assumption of function, particularly for individual mQTL results requires caution. mQTL driven CpGs are proposed to be in loci that are under less evolutionary or functional constraint in comparison to constituently hypomethylated CGI loci [190].

The extensive and replicable genetic influence on DNAm, therefore, needs careful consideration, as larger and meta-analysed datasets are now being evaluated. The functional informativeness of mQTLs needs to be balanced by their ubiquity and mechanism of detection. Therefore, a recontextualization of the functional implications of mQTLs is needed in the same light as has occurred for eQTL [180]. This is for three major reasons, firstly, the scale of the number of potential mQTLs needs to be fully acknowledged beyond the narrowly focused, albeit robust, array CpGs. The 450 k and EPIC v1 platform analyses have estimated that ~ 34–45% of the CpGs on these arrays are under genetic influence [185, 191]. These arrays at maximum are only assessing ~ 3% of the CpGs in the DNA methylome, although this selection is functionally enriched. If these levels are extrapolated out to the rest of the genome, > 10 M CpGs at least are under genetic influence. This underestimation of population genetic variation’s ability to shape the DNA methylome also needs to acknowledge that array results are excluding or do not assay the many obligatory or potential facilitative DNAm differences that are driven by larger genetic variants (CNVs, STRs) or compound haplotypic effects [167, 192]. So, due to these ubiquitous genetic influences, the interpretation of the likelihood of substantial functional impact for an individual CpG or mQTL must be circumspect and bear in mind the sizeable non-assessed DNA methylome.

Secondly, equal caution is needed in the correlation of DNAm to gene expression beyond a mere statistical association with any gene within a defined large window, e.g., 1 Mb. This is the same restraint as needed for SNP to gene associations, in that the simple assumption that the nearest gene is not always implicated. The a priori likelihood of a mQTL influencing expression, i.e., that its associated CpG is a quantitative trait methylation locus, eQTM, needs to be considered. Regional DNAm changes have increased likely function compared to an individual CpGs. Also, further support is needed that the correct target gene been identified, i.e., is the co-location of this CpG within this gene’s promoter, or tissue-specific enhancer from chromatin segmentation data, and if the latter, is there supportive interaction data with this distal CRE and the appropriate promoter from Hi-C data. Furthermore, whilst statistically significant, the direction of effect may not be biologically correct, as even within CGI promoters classic hypermethylation is not always associated with reduced expression [88]. Also, proposed changes in gene body methylation influencing expression need functional data to support them.

Thirdly, multiple variants can be in LD with a GWAS SNP, and all of these can influence the DNAm of multiple CpGs, as mQTLs, through both biological (obligatory and facilitative) and technical effects (e.g., see DNAm array influences below). However, the significant correlation between the GWAS-SNP and DNAm does not demonstrate that this is in fact the functional mechanism, as this requires experimental evidence, such as CRISPRi or CRISPR-activation (CRISPRa) in the appropriate pathogenic tissue and/or stimulated state.

### Mendelian randomization for the functional exploration of DNAm

As one method to assess function, the impact of DNAm was quantified on gene expression in multiple complex traits through a multivariable Mendelian randomization (MVMR) framework [193]. This was performed by integrating eQTL and mQTL and the role of *cis* transcripts in mediating a DNAm to complex trait causal relationship. The DNAm of the *PARK7* promoter CpG, cg10385390, through its influence on reducing expression was found to increase the risk for inflammatory bowel disease. Also, this study identified DNAm of cg13428477 increased *PDIA5* expression and this was associated with increased platelet count. *PDIA5* also contains an intragenic HSM [167] associated with the overlapping GWAS lead SNP, rs3792366.

### Genetic influences on active and repressive chromatin peaks are also abundant

Genetic influence on histone PTMs have also been explored, as in the previously described GTEx H3K27ac hQTL analysis [126], although these analyses are impeded numbers wise by the requirement for tissue samples for ChIP or similar method. An additional analysis for genetically associated H3K4me1, H3K4me3, and H3K27ac peaks (hQTLs), both local (± 2 kb of peak boundaries) and distal (< 2 Mb) was performed in 75 lymphoblastoid cell lines [194]. This study identified ~ 22 k (H3K4me1), ~ 14 k (H3K27ac), ~ 9.5 k (H3K4me3) hQTLs and that these were enriched for SNPs associated with autoimmune diseases. Integration of H3K27ac peaks from an SLE case control lymphoblastoid dataset with GWAS data, identified acetylation was enriched on disease-risk haplotypes that influenced local gene transcription [195]. The Blueprint consortium analysis in three major blood cell types (CD14^+^ monocytes; CD16^+^ neutrophils; and naive CD4^+^ T cells) identified that ~ 43.3% of eQTL sentinel variants were also associated with H3K4me1 or H3K27ac hQTLs (i.e., identical or in high LD, r2 ≥ 0.8) [68].

## Epigenome-wide association studies (EWAS)

### Analysis of the DNA methylome for association with complex traits

The inherent stability of DNAm has empowered Epigenome-wide Association Studies (EWAS) at scale. This DNA methylome analysis aims to identify specific Differentially DNA methylated Positions or Cytosines (DMP/Cs) related to a trait or phenotype. As well, clustered co-directional variation in CpGs can be statistically defined as DMRs, and these, as mentioned, due their increased potential for regulatory effect, are enriched for function [83].

### DNAm array analyses have enabled a population-scale assessment of the DNA methylome

Genome-wide DNAm arrays, whilst assessing a comparatively limited number of the total CpGs in the genome (i.e., Illumina EPIC v2.0 935 k, ~ 3%) still maintain two critical advantages over WGBS and, thereby, continue to perform an extremely useful role for the epigenomics field. Firstly, a known set of CpGs, present on the array, will be consistently assayed and, secondly, highly robust, and equivalent data is obtained for these CpGs with this methodology. These two factors have enabled high-throughput analysis of large-scale epidemiological DNA datasets. The precision of these DNAm array data, estimated to be equivalent to ~ 100X sequencing depth [196], has supported multiple EWAS analyses [197, 198], the novel discovery of a pan-tissue DNAm ‘clock’ and the subsequent research into phenotypic or ‘biological’ DNAm ‘clocks’ [199] (detailed further below), as well as the construction of predictors of plasma protein levels, termed ‘EpiScores’ [200].

Significantly higher read depth requirements are needed for the quantitative assessment of DNAm in comparison to genomic sequencing, which by contrast is generally focused on the germline identification of only three discrete categories: common homozygote, heterozygote, rare homozygote. Whilst the initial coverage level of 30X for 2nd Gen-seq was recommended for the NIH Roadmap Epigenomics Project, this was subsequently recognised as inadequate, with WGBS saturation analysis experiments estimating a minimum of 85X is required for DMC calling [201]. Targeted DNAm sequencing approaches aim for substantially deeper resolution (e.g., 100X [202], 300X [203], etc.). Furthermore, in contrast to the array data, 2nd Gen-seq suffers from stochastic coverage across all the CpGs in the genome. Therefore, individual CpGs will vary drastically away from the quoted mean or median coverage, and this increases or decreases the quality of result for that specific CpG in that specific sample. Consequently, whilst DNAm trait estimators and clocks can be constructed via WGBS, their portability from the initial findings is limited due to this inability to cover the same CpGs in subsequent analyses with reliable data. WGBS analyses, in fact, often end up covering a vastly smaller number of CpGs than the total DNA methylome, once even moderate coverage thresholds are employed across all the samples of a particular analysis, and this smaller ‘well-covered’ set will differ even in technical replicates.

Hence the epigenomics community has continued to use and benefit from DNAm array technology, and the release of a mouse [204] and an updated human array EPIC v2 (935 k) [205] in the last 2 years attest to the continued enthusiasm for this platform. A high-throughput screening DNAm human array is also in the pipeline for future release.

### Delineating six classes of genetic influence on DNA methylation array analysis

**Fig. 3:**
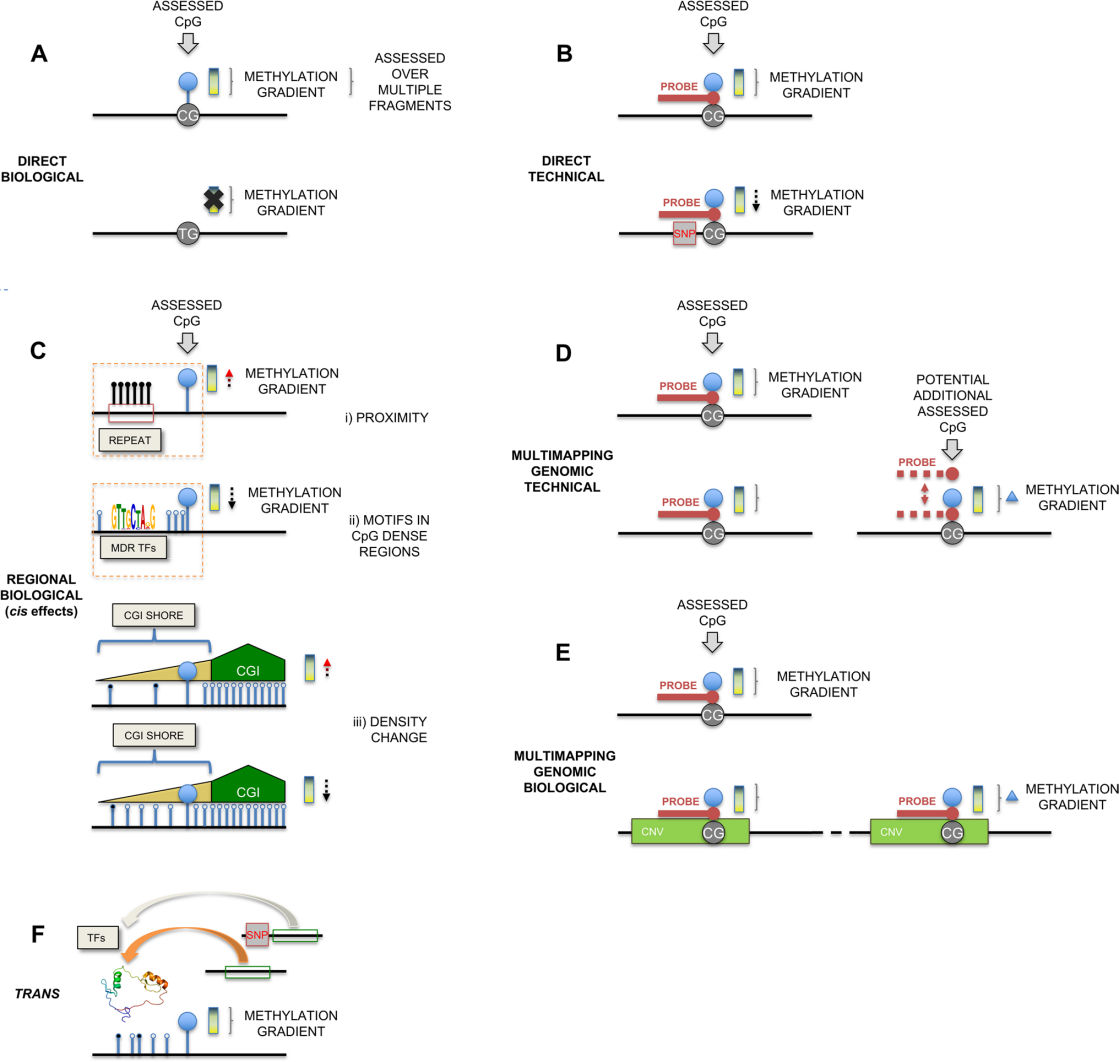
Genetic influence on DNA methylation assessment. **A** Direct Biological. A CpG-SNP leads to the loss of DNA methylation ability at the interrogated cytosine. **B** Direct Technical. A genetic variant residing under the CpG probe interferes with binding. **C** Regional Biological. Cis-effects due to genetic variation (Repeat Elements, Methylation Determining Regions/MDRs, CpG density). CGI = CpG Island. **D** Multimapping Genomic Technical. CpG probe binds inadvertently to multiple locations. **E** Multimapping Genomic Biological. CpG probe binds to multiple sites due to CNV encapsulating interrogated CpG. **F**. Trans effects. Genetic variation modulates the expression of distal TFs influencing their binding to the interrogated locus (Adapted from [207])

For DNAm array-based analysis the considerable influence of genetics affecting the DNAm quantification either strongly or subtly can be broken down into six major classes (Fig. 3): (A) Direct biological—due to genetic mutation of the CpG dinucleotide itself, i.e., an allelic discordance with the CpG present on one allele and not the other, commonly a SNP within the CpG site (a CpG-SNP). This will present with an extreme trimodal DNAm pattern, if the CpG is fully methylated (i.e., ~ 0, 50, 100%); (B) Probe technical—due to genetic variation most likely directly under the array probe (a 50 bp complementary sequence) that reduces the likelihood of binding of the probe to that specific allele. In this case, there is under-representation of the DNAm measure of this non-binding allele and if there is actual biological variation in the DNAm state between alleles then this will be reduced or missed completely; (C) Regional biological—these are genetic *cis*-effects that vary between haplotypic states, for example, if the assayed CpG is (i) proximal to polymorphic TFBS that influences DNAm state of a CGI (MDRs [164]), (ii) a polymorphic heavily methylated repetitive element that influences the DNAm of the locus making the CpG prone to be slightly more methylated, or (iii) polymorphic CpGs in phase that influence the local neighbourhood CpG density. Any SNP in strong LD with any of these genetic factors, such as the inclusion of this repetitive element on this haplotype, would be observed to be a mQTL; (D) Multimapping Genomic Technical—due to the ability of the probe sequence to bind to more than one unique region of the genome. The resultant assayed CpG will be an average of these multiple locations. This DNAm average will depend on the likelihood of binding to the sequences and regional properties of the various loci as well as potential stochastic binding effects; (E) Multimapping allelic variation biological—if the probe resides within a region that exhibits an unknown CNV, the DNAm signal will not only increase in intensity [206], but will also be the average of the CNV methylation states. Consequently, any disparity in the DNAm between the copies will be an influence; and finally, F) Genetic *trans* biological—*trans* effects on the locus, e.g., the assessed CpG is within or near a TFBS that via the action of a TF binding changes the local DNAm. If the expression of this TF is itself under genetic influence by a SNP, then this SNP will be a *trans*-mQTL for the assayed CpG.

Loci can be under the influence of a number of these contributing genetic factors to a lesser or greater degree, and in powerful DNAm studies some of these more subtle effects will become consistent enough above technical noise to be readily detected by the highly accurate array technology. This multitude of genetic effects contribute to the strong population influences observed on DNAm data [208].

### Heterogeneous DNAm signals can be deconvoluted in cellular fractions

DNAm data, due to its cell-type specificity, provides information on the constituent cell types present within a heterogeneous sample. As blood-derived DNA is representative of the various leukocytes present when the DNA was extracted, this allows cell-type deconvolution to be performed utilising leukocyte subtype-specific DMCs [209]. Multiple tissue-type algorithms have been constructed to assess the proportion, cellular infiltration, or contamination of samples. These are highly informative for interpreting the resultant measured DNAm variation [210]. A more specialised higher resolution of cell type may be required in some cases and this deconvolution can bring further insights into pathogenic processes, e.g., the macrophage subtypes M0, M1, etc. [211].

However, these increasingly detailed epigenomic data do add further complexity, as the definition of cell type is not yet fixed. An evolutionary stance is proposed as a tool to help in the definition of cell type identity and with this the concept of a cell’s ‘core regulatory complex’ (CoRC) of transcription factors [212]. Also, whilst there is a clear mechanism for DNAm mitotic inheritance to maintain the cellular epigenomic signature, for chromatin PTMs this is only most strongly supported for repressive signatures, such as H3K9 methylation, and to date this has only been observed in yeast models [213]. Although, recycled parental histones are observed to remain close to their genomic location through DNA replication, potentially hinting at transfer of this epigenetic information [214].

### Robust and replicated DNAm associations have been identified for common diseases

EWAS via DNAm arrays (or DNAm wide association study: MWAS) have been highly successful with > 1000 studies detailing numerous associations listed on the EWAS Atlas [215]. The first studies were performed by 27 k arrays [216, 217] and have subsequently progressed with the technology updates. Robust statistical thresholds have been proposed for the array platforms (e.g., p < 9 × 10^–8^ for EPIC v1) as well as study size impacts on power (e.g., n ~ 1 k samples) [218]. Most analyses have employed peripheral blood-derived DNA due to its availability, and as such many of these findings are biomarker associations, having not been performed in the appropriate pathogenic tissue for the disease or trait. For example, consistent blood DNAm associations with the obesity phenotype of BMI include many overlapping findings for components of the metabolic syndrome, comprising hyperlipidaemia, hyperglycaemia, and inflammatory measures [207]. These epigenetically derived data are still physiologically informative, as a blood DNAm estimator of BMI, constructed by elastic net regression, was able to calculate ~ 32% of the variance in BMI and this measure was better than actual BMI in predicting poorer metabolic health [219]. Previous comparisons between DNAm predicted and genetic polygenic risk scores (PRS) have shown that these are essentially independent and additive in nature, e.g., for BMI predictors: DNAm 12.5%, PRS 10.1%, and combined 19.7%. However, this is starkly different for smoking where almost all the strong predictive power is via DNAm at ~ 60.9%, with PRS only 2.8%, and combined at 61.4% [220]. DNAm associations have been recently formulated into DNAm profile scores (MPS) in a similar fashion to PRS [221]. Further supporting the interconnected nature of blood DNAm adiposity predictors, six body-fat-related traits were evaluated and these DNAm correlations were observed to be of greater magnitude than their genetic counterparts [222]. Furthermore, a small number of 8 CpGs correlated with sex-specific BMI. The dramatic physiological intervention of bariatric surgery has been explored epigenetically in 40 severely obese individuals before and 1-year post-surgery, with 4857 DMCs identified in peripheral blood. Of these ~ 51% were directly attributable to the change in BMI observed [223].

A peripheral blood T2D meta-analysis performed in five European cohorts identified 76 CpG sites associated with incident T2D from 1250 cases and 1950 controls, with 64 of these then replicated in a South Asian Indian population [224]. This included DNAm associations within *ABCG1*, *TXNIP*, *SREBF1* and *CPT1A*. As with the BMI findings, many of these are overlapping metabolic syndrome phenotypes, including hyperlipidaemia, hypertension, and obesity. In fact, these results were primarily driven by BMI, with 33 CpGs directly associated, and after adjustment for this phenotype, only four CpGs remained significant. This reduced to three after an adjustment for smoking status. These four included the two most significant CpGs, *TXNIP* (cg19693031), previously associated with sustained hyperglycaemia [225], and *ABCG1* (cg06500161), with multiple metabolic associations [207], as well as *CFL2* (cg21234053), although this has been associated with maternal smoking previously [226]. However, further DMCs were still able to be identified in a recent US study in African- and European-Americans with seven novel findings in the former and three in the latter [227]. Intriguing functional insights into the long-term effects of hyperglycaemia have identified glucose increasing the post-translational modification, O-GlcNAcylation at S878, of DNMT1, inhibiting this maintenance enzyme in the liver, leading to downstream alterations of the epigenome [228].

Cardiovascular disease (CVD) traits have been explored via EWAS, such as ischaemic stroke [229]. In the Framingham Study, biomarkers of CVD were identified through ML by combining DNAm and RNA data in a decision tree, light gradient-boosting machine (LightGBM) [230]. These were subsequently confirmed in isolated monocytes. Bespoke predictors for specific diseases are likely to be superior to more generalised phenotypic measures [231] and for CVD, a DNAm-based composite predictive biomarker, DNAmCVDscore, was built from an Italian cohort for short-term CVD events [232]. This was then validated in four further European cohorts. This score included DNAm estimators calculated from ML elastic net regression for the contributory disease-related factors of BMI, blood pressure, fasting glucose, insulin, cholesterol, triglycerides, and coagulation. This DNAmCVDscore outperformed the SCORE2 clinical algorithm that measures 10-year CVD risk. Additionally, a myocardial infarction (MI) blood DNAm predictor was constructed in ~ 11.5 k coronary heart disease (CHD) free individuals across nine studies (US and EU) under the umbrella of the CHARGE consortium [233]. 52 CpGs were associated with future MI risk, and these were noted to include those residing in genic loci involved in calcium regulation (*ATP2B2*, *CASR*, *GUCA1B*, and *HPCAL1*).

Respiratory studies have included asthma EWAS, identifying, as predicted, signals strongly driven by an increase in eosinophils [234]. Functional exploration in primary airway epithelial cell cultures of the functional effect of type 2 cytokine IL-13, a key mediator of allergic airway diseases, identified induction of widespread and long-lasting changes to the airway epithelial DNA methylome [235]. Recent work has explored Idiopathic Pulmonary Fibrosis (IPF) through a high-resolution EWAS, involving a myeloid-specific deconvolution of the pathogenic cell-type, the airway macrophage [211]. Another study evaluated DNAm derived from nasal brushing, identifying that only three CpGs were needed for a strong predictor of childhood allergy diagnosis [236]. These changes were associated with an influx of T cells and macrophages, indicative of allergic inflammation.

The direct analysis of neurological disease is clearly limited by the confines of the brain, however, any surrogate biomarkers for Alzheimer’s or Parkinson’s disease or other disorders that can be identified would have substantial clinical utility. Post-mortem analyses have been performed to give potential insights into disease pathogenesis, with, for example, a meta-EWAS across the cortex for Alzheimer’s disease identifying ~ 1.5 k DNAm changes associated with Braak stage and 220 cross-cortex DMCs with neuropathology [237]. A frontal cortex EWAS has identified specific DNAm changes for dementia with Lewy bodies, revealing novel pathways involved in this disorder [238].

### Array analysis of the blood DNA methylome can identify the prevalence of multiple common diseases in combination

The ability to assess disease risk across a whole combination of illnesses through blood based DNAm array (EPIC v1) EWAS was assessed in ~ 18 k individuals from the Generation Scotland cohort [239]. Cross-sectional prevalence of 14 common diseases identified 69 DMCs (58 novel) associations for breast cancer (10), chronic kidney disease (1), ischemic heart disease (6), and T2D (52). The longitudinal incidence of 19 disease states identified 64 DMCs (56 novel) that associated with the incidence of Chronic Obstructive Pulmonary Disease (6) and T2D (58). However, replication of previous studies was lower than expected, indicating that increased consensus is needed for future large biobank analyses regarding statistical rigour, phenotypic definitions, as well as reporting standards [239].

A powerful meta-analysis across ~ 22 k individuals with Illumina DNAm array data explored the subtle shifts in 12 leukocyte cell blood types and their association with exposures, phenotypes, and disease [240]. A validated leukocyte reference was constructed using flow-cytometric count and sorted cell WGBS data. Differing leucocyte proportions were associated with age and sex, as well as smoking and obesity. Independently of major epidemiological risk factors, follow-up data identified that an increase in naïve CD4^+^ T-cells correlated with reduced risk of all-cause mortality.

### Histone tail modification association studies have also been performed for common diseases

EWAS using chromatin PTMs, for example, Histone Acetylation-wide association studies (HAWAS) interrogating H3K27ac peaks, have been performed, but in a much more limited scale, to date, due to the issues of retaining robust chromatin data. These have included a HAWAS for autism spectrum disorder (ASD) in 257 post-mortem case–control samples that identified ~ 5 k CREs associated with diagnosis [241]. A similar HAWAS for Alzheimer’s Disease in 47 post-mortem entorhinal cortex samples identified ~ 4 k differential peaks [242]. An HAWAS in immune cells for *Mycobacterium tuberculosis* infection identified > 2 k CREs in a small discovery set of Singapore Chinese (n = 46), with replication across populations in South Africans, where the fold-change direction was concordant for 86%. As previously discussed, these sequencing-based enrichment analyses performed in population-based samples need to clearly delineate between signals that are driven genetically from pure epigenetic variation, particularly due to the potential impact of population variation in CNVs, Indels and STRs. Exploring SNP genetically driven peaks, the ASD HAWAS identified ~ 2 k haQTLs within the general population [241].

## The ageing epigenome

### The DNA methylome changes over a lifetime with both stochastic and consistent components

Ageing is an obvious but significant risk factor in ageing-related diseases. The biological ageing mechanism itself is extremely rigidly controlled, as no extreme outliers of distinct species expectations have ever been observed. Understanding the molecular drivers of ageing may however bring novel insights into the pathogenesis of ageing-related diseases, and epigenetic changes are recognised as one of the hallmarks of the ageing process [243]. Ageing changes in the epigenome were first documented in salmon in the 1960’s [244]. The epigenomic landscape is thought to deteriorate over time in a process termed ‘epigenetic drift’. A landmark study for this model was the identification that even comparing between monozygotic (MZ) twins, de facto genetic clones, a DNAm assay increasingly diverged with age [245]. The advent of high resolution and high throughput DNAm arrays then enabled further intricacies to be discovered. Ageing-related increases in DNAm were found to be enriched in promoters of polycomb group target genes [246], as well as in bivalent domains that possess both active (H3K4me3) and repressive chromatin (H3K27me3), which are frequently found in developmental gene promoters [247]. These promoters possessing age-related DMRs also commonly become hypermethylated in cancer [248].

Of note as well, this ageing-related DNAm promoter hypermethylation, as in the case of the *ZNF577* promoter [248], which has also been observed in cancer, may also be reflective of the detection of the altered epigenetic state of low-level somatic clones. This *ZNF577* promoter hypermethylation has also been observed with the ageing-related polycythaemia vera (PCV) *JAK2* V617F pathognomonic mutation [249].

As previously mentioned, mitotic rate is a driving factor in hypomethylation due to inconsistencies in the fidelity of the remethylation process, with late-replicating sparse WCGW CpGs particularly venerable [113]. Ageing-related loss of DNAm also occurs within TEs subfamilies. These may contain lineage-specific TFBSs, leading to alternate lineages become active and subsequently this ageing-related loss of lineage maintenance may predispose to malignancy [250]. DNAm changes may also modulate TE roles in chromatin organisation and heterochromatin homeostasis [141].

Early replicating regions of the cancer epigenome are seen to be prone to hypermethylation [251], and in parallel an ageing-related enrichment for hypermethylation is seen in the early replicating, as well as highly transcribed, tRNA loci [252]. This DNAm change was observed for certain tRNA copies, and in further parallels, this isolated tRNA hypermethylation has also been seen in the cancer methylome, with subsequent functional effects on tRNA transcription [253].

The influence of local CpG density on ageing was further explored in a peripheral blood longitudinal analysis of 450 k DNAm array data, with ~ 346 k robust CpGs evaluated, in ~ 600 elderly samples (67–80 years) [254]. Ageing changes were detected in > 50% of CpGs. These were not driven by major cell type variation and were most strongly evident at ~ 8 k CpGs residing within low CpG density and heterochromatic loci. These cytosines lack the reinforcement in DNAm state provided by neighbouring CpGs that aids DNMT efficiency [255] A quarter of these (~ 1.5 k) were under strong genetic polymorphism influence by variants that altered the CpG density of the region and/or bases adjacent to CpGs [254]. DNAm ageing trajectories were more variable at low CpG density regions with increased variable changes in DNAm with age in older adults (> 65 years), but a directional loss of DNAm across the midlife age range (≤ 65 years). By contrast, dense CpGs can recruit CXXC proteins (including H3K4 methylators: CFP1, MLL1, MLL2, and DNA demethylase: TET2) that more strongly reinforce their low DNAm status [254].

### DNAm ‘clocks’ are influenced by immunosenescence, but also capture ageing-related stochastic changes within different genomic functional units

As mentioned above, DNAm arrays expediated the discovery of DNAm ageing ‘clocks’, with the paradigm shift coming from a pan-tissue clock that was able to be constructed, using the same CpGs that worked across different tissues [256]. Therefore, this implied that epigenetic signals existed beyond those driven by recognised ageing-related tissue-specific cellular proportional changes, as well as the random processes associated with drift. A consistency existed in the directionality of DNAm changes within certain genomic units, which occurred steadily enough across cell-types that it could estimate age within ± 3 years [257]. Furthermore, in this error, a component of ‘biological age’ was captured, as positive epigenetic ‘age acceleration’ correlated positively with morbidity and mortality [258]. Whilst later dissection of this effect has identified that a small fraction can be contributed to changes in rare cell types, since the training set was predominantly blood derived and as well that blood cells infiltrate many tissues. Furthermore, cellular compositions themselves are also related to mortality [240, 259]. Immunosenescent T cells (CD8^+^CD28^−^), and in general the activation of T cell and NK cells [260] may contribute to this observation. Although, the pan-tissue concept is still valid [257]. However, it should also be noted that these few hundred CpGs present within the defined Horvath clock (353 CpGs) are not extraordinary in themselves, but merely good representatives that work well together to capture the directional changes that occur stochastically within different genomic units. Other sets of CpGs can be selected that can equally capture these changes [261]. Clock CpGs are well represented in CpG dense functional loci [231]. They are capturing the multiple systemic ageing processes, estimated to comprise at least twelve distinct modules [262].

A recent analysis of immune epigenetic ageing across six major purified immune cell types (naive B, naive CD4^+^ and CD8^+^ T cells, granulocytes, monocytes, and NK cells) via EPIC v1 DNAm arrays identified only a small, although statistically enriched fraction was consistent across all cell types [263]. However, whilst benefiting from isolated cell-type analysis, the sample size was only 55 (ranging 22–83 years). Of interest are those consistent changes that were able to be identified, such as in promoter region of *RCAN1* and *KLF14*, although most changes that were shared across all cell types were moderate. Though, *ELOVL2*, previously identified as a strong outlier of cross tissue ageing-related hypermethylation [264] was also supported in this analysis. Opposing changes were also seen in differing cell types, for example, T cell specific differences in a *BCL11B* gene body CpG (cg27123256), increased DNAm with age in naive CD4^+^ T cells and decreased in non-T, and a *FAM19A1* gene body CpG (cg03530364) increased DNAm in non-T cells but decreased in CD4^+^ T cells. Increased variability at certain CpGs has also been observed previously with age, and was proposed to be associated with fundamental ageing pathways [265].

### DNAm ‘clocks’ are predictors of chronological as well as phenotypic biological age associated with ageing-related diseases

Once it was recognised that aspects of ‘biological’ age could be captured by DNAm clocks, efforts then focused on improving this measure, leading to the construction of ‘phenotypic’ clocks. Firstly, the PhenoAge clock comprised DNAm surrogates of the NHANES PhenoAge biochemical and haematological data, including albumin, creatinine, glucose, C-reactive protein (CRP), lymphocyte percent, mean red cell volume, red cell distribution width, alkaline phosphatase, white blood cell count and age [266]. Following this, the GrimAge clock was constructed using DNAm estimates of seven plasma protein levels: adrenomedullin levels, beta‐2 microglobulin, cystatin C, growth differentiation factor 15, leptin, tissue inhibitor metalloproteinase 1, plasminogen activation inhibitor 1 (PAI-1), plus crucially a quantitative smoking estimate (PackYears) as well as a DNAm age estimate [199]. This clock possessed the best prediction, at that time, of future cancer or cardiovascular disease as well as mortality [267]. This was recently updated to GrimAge2, with the addition of the long-term glycaemic control measure haemoglobin A1c (HbA1c) and inflammation with CRP [268]. A further expansion on this is ‘bAge’ from the Generation Scotland dataset, that incorporates the GrimAge measures with age and sex plus 109 EpiScores for ageing-related plasma proteins [269].

DNAm clocks have been correlated with a plethora of ageing-related diseases [257]. For example, an analysis of future T2D risk with GrimAge clock in the CARDIA study (n =  ~ 1 k) revealed that in obese individuals a positively accelerated GrimAge was associated with an odd ratio of ~ 2.57 for the 10-year risk of developing T2D [270].

## The environmental influence on epigenome

### The tobacco DNAm biomarker hints at substantial potential to quantitate external exposures

The analysis of the epigenome has been postulated as a molecular measure to quantitate non-genetic environmental influences on disease [271]. The standout signature in peripheral blood DNAm of tobacco smoke intake and/or exposure levels strongly indicated this potential [272]. This signal is robustly observed between discordant MZ twins [273]. Hypomethylation of the top CpG, *AHRR* cg05575921, is a predictive biomarker of future lung cancer [274], with even proposed utility in population lung cancer screening [275].

Whilst the tobacco-associated DNAm changes are one of the strongest effects that can be observed from peripheral blood, they do in fact vary across specific blood-cell subtypes and are an excellent example of how cellular heterogeneity influences the epigenetic signal. These DNAm signatures are influenced in tandem by the leukocyte proportional changes as well as intracellular DNAm changes. For example, the *AHRR* cg05575921 signal is more strongly represented in the myeloid lineage, which itself increases in proportion due to smoking [276]. Thus, this combined effect leads to this specific DNAm biomarker being so prominent. By contrast, the tobacco-related hypomethylation of *GPR15* cg19859270 is lymphocyte specific [277]. Furthermore, regarding cellular proportions, smokers are observed to have a 7.2% reduction in naïve B cells (CD19^+^) [278].

### DNA methylation associations with pollutant exposures may bring novel insights to disease aetiology

The influence of long-term ambient air pollution exposure (1-year average home outdoor concentrations) on atherosclerosis was explored via EWAS. This was performed in CD14^+^ monocytes, a cell-type critical in atherosclerosis pathology, via fine particulate matter collectively termed PM2.5 and Oxides of nitrogen (NOX) in the Multi-Ethnic Study of Atherosclerosis (MESA) study (n ~ 1.2 k) [279]. This identified four DMRs for PM2.5 in or near to *SDHAP3*, *ZFP57*, *HOXA5*, and *PRM1*, and two DMRs for NOX also at *SDHAP3* and *ZFP57*. The *HOXA5* DMR was associated with monocyte expression of *HOXA5*, *HOXA9*, and *HOXA10*. These epigenetic changes may be insightful as to how air pollution contributes to cardiovascular disease risk.

Associations of annual ambient PM2.5 components have also been explored with DNAm phenotypic clocks [280]. An analysis in ~ 700 elderly men identified PhenoAge clock acceleration measured against a 1-year moving average from a local air-quality monitor. The difference between the interquartile range (IQR) in PM2.5 levels led to an increase of 0.16 years in DNAm PhenoAge acceleration. Of note, an analysis of the contributing chemical components of PM2.5, identified that a similar IQR increase in specifically the lead or calcium component caused an even larger increase of 1.45 and 0.62 years in DNAm PhenoAge acceleration, respectively.

Arsenic exposure at low exposure levels in water and food is related to multiple health outcomes, including cardiovascular disease (CVD) and hypertension [281]. It can also induce epigenetic modifications in experimental exposure models. A prospective EWAS was performed in ~ 2.3 k American Indians with blood DNAm data analysed with urinary arsenic species, measured by high-performance liquid chromatography and plasma mass spectrometry. Arsenic-associated DMCs at 20 and 13 CpGs were associated with CVD incidence and mortality, respectively. Functional models of arsenic-induced atherosclerosis also support the hypothesis that diabetes and redox signalling are involved in its pathogenesis [281]. Arsenic exposure also leads to phenotypic DNAm clock age acceleration [282].

There is clear utility in these exposure DNAm biomarkers for epidemiological measures [283], but additionally functional insights may also be possible in appropriate pathogenic tissue through delineation of how the DNAm measures are being influenced by cellular subtype proportions, DNA sequence, and TF binding [284].

## Advancing analysis of the DNA methylome

### Epigenomic analysis will be advanced with increasing direct long read DNA data

The analysis by single-molecule or 3rd Gen-seq technologies, such as ONT and PacBio, is enabling substantial novel insights by the generation of direct genetic and DNA modification long read data [285]. These methods are bringing new knowledge to well-studied epigenetic phenomenon, with ONT analysis detecting 42 novel imprinted DMRs in the human genome [286], as well as enabling improved assessment of skewing in X‑chromosome inactivation (XCI) [287], respectively. Inaccessible regions of the genome, such as centromeres, have now been able to be surveyed for their DNA modification state [72].

Additionally, improvements in accuracy and in the ability to scale up ONT long-read analysis will aid large-scale haplotypic integrative analyses, by incorporating SNP, InDel, CNV, STR, and Structural Variant (SV) assessment in tandem with DNA modifications [288]. This will support deeper evaluation and regulatory understanding of the substantial common disease related HSM already observed [167]. CpG-SNPs are obligatory mQTLs and common CpG-SNPs are excluded by design from DNAm arrays, or those inadvertently included are routinely removed from analysis. However, when multiple CpG-SNPs are in phase these can contribute strong population DMR effects [167]. A recent Human Genome Structural Variation Consortium (HGSVC) analysis of 32 human genomes by a combination of long-read PacBio and Strand-Seq was evaluated for SVs in LD with GWAS SNP and identified 4677 unique trait-associated SVs [289].

Long reads also enable methodologies, such as nucleosome occupancy and methylome sequencing (NOMe–seq), to determine the long-range phasing of dynamic, tissue-specific, and allele-specific regulation on identified disease-related haplotypes [290]. In this analysis, accessible DNA is marked by a recombinant DNA methyltransferase *(M.CviPI)* for GpCs not endogenous CpGs. The integration of long read data is starting to rectify the weaknesses of array focused as well as short-read 2nd Gen-seq analyses and enable a more nuanced evaluation of haplotypic genetic and epigenetic effects. Furthermore, previously unrecognised subtle patterns are now visible in these single molecular resolution data, with nucleosome periodicity clearly signalled in DNAm, and contributing to DNAm heterogeneity in bulk analyses [291].

### DNA methylome data will dramatically increase with the shift to 3rd generation sequencing

Whilst rare imprinting disorders and monogenic diseases, such as FXS, have been longstanding clinical genetic pathology tests requiring DNAm analysis, additional risk factors for common disease will be recognised with the increased availability of DNA modification data. This will arise as the large biobank datasets, as well as pilot neonatal whole genome sequencing currently being embedded, in future likely shift to direct molecule 3rd Gen-seq technologies. Furthermore, the availably of this scale of data will stimulate improved statistical approaches for assessing variation in the DNA methylome, such as novel Bayesian methods [292, 293].

### Real-time DNAm diagnostic utility for CNS tumours

DNAm dysregulation is routinely observed during tumour development. These changes can be used to profile as well as pinpoint therapeutic vulnerabilities through the identification of druggable targets for sites with *cis* acting aberrant DNAm [294]. There is immense diagnostic potential through the precision provided by epigenomic assessment of tumour biopsies and in the determination of cancer of unknown primary [295]. Through the implementation of a ML random forest algorithm, a DNAm-based classifier was devised for the ~ 100 central nervous system tumour types [296]. A prospective evaluation of this diagnostic tool found that in ~ 1 k samples, ~ 15% were histologically ambiguous but that a classification based on DNAm was possible. For ~ 12%, a change in diagnosis was called, and for ~ 93% of these discordance calls, after retrospective assessment with additional histological and genomic analysis, the DNAm tool was deemed to be correct. This DNAm classifier was subsequently used successfully for rapid assessment via ONT, enabling intra-operative surgical decisions, clearly indicating the increasing clinical utility of epigenomic analysis for pathological diagnosis [297].

### A potential DNAm biomarker predicts acute lymphoblastic leukaemia risk from birth

Analysis of the DNA methylome by Illumina EPIC v1 DNAm array derived from the archived neonatal blood spots from MZ twins discordant for paediatric acute lymphoblastic leukaemia (ALL) (n = 41 pairs) revealed 240 DMCs and 10 DMRs consistently associated with ALL risk [298]. The top four DMCs were validated by methylation-specific droplet digital PCR, with a region overlapping *TRIM39*-*RPP21* being most significant. These changes may be a potential biomarker of future ALL risk with a predisposed epigenome already present at birth.

### Clinical utility of EpiScores

The quantitative EpiScores for internal circulating plasma proteins [200] potentially benefit from being more accurate measures of long-term trends, with, for example, the DNAm estimator for CRP outperforming serum levels as a biomarker of chronic inflammation in relation to brain ageing [299].

A composite predictor from 45 DNAm EpiScores for cardiovascular disease was successful in CVD risk evaluation independent of the clinical prediction algorithm (ASSIGN) and cardiac troponin I levels [300]. Similarly, EpiScores were effective for T2D 10-year incidence prediction beyond standard risk factors [301].

Additional DNAm clinical utility can come from assessing therapeutic decision making and pharmacological monitoring. An exemplar of this is monitoring the response to dexamethasone, due to its immunosuppressive potential, in glioma treatment. A neutrophil dexamethasone DNAm index (NDMI) was constructed that was a more precise monitor than blood analysis for glucocorticoid response in glioma survival [302].

## Therapeutic modulation of the epigenome through epidrugs

### Epidrugs are providing novel therapeutic avenues with considerable potential

The successful therapeutic targeting of the cancer epigenome through the broad DNMT inhibitor (DNMTi) Azacitidine was first introduced in 2004 for the treatment of myelodysplastic syndrome [303]. This cytidine analogue leads to global DNAm reduction, including the removal of hypermethylation at tumour suppressor genes, with additional indirect effects mediated by reprogramming, and, furthermore, due to direct incorporation into DNA, is cytotoxic [303]. Other reprogrammers, such as Histone Deacetylate Inhibitors (HDACi), EZH2 inhibitors (EZH2i), and inhibitors of BET (BETi) binding to acetylated histones, also found subsequent success as oncological therapeutics, with multiple proposed actions including induction of cell cycle arrest and repression of accelerated cell growth. This applicability of these epigenomic modifiers was made clear through the large-scale cancer sequencing projects that identified that mutations in the epigenomic machinery were frequent, as with developmental orders, with ~ 50% of human cancers possessing mutations in chromatin organisation enzymes [303]. However, modulation of the epigenome has considerable potential for non-malignant diseases as well, with potential for BETi and HDACi in vascular inflammation and atherosclerosis, respectively [55]. The wide-ranging therapeutic possibilities of H3K27 demethylase inhibitors involve KDM6A/B inhibition as an anti-inflammatory mechanism for autoimmune and inflammatory disorders, as well as an immunopathological suppressant for infectious pathogens [304]. Additionally, the exciting future potential beyond these broad modulators is precise genomic targeting of epigenomic activators or inhibitors, through CRISPRa or CRISPRi, respectively [305]. The tethering of epigenomic writers or eraser enzymes to an inactive Cas9 with guide RNA enables locus targeting of CREs to modulate gene expression levels [306]. Functional screens can also benefit greatly from this technology, for example, by targeting specific promoters within a gene to identify the critical pathogenic isoform [307]. The ability to target specific alleles through placing SNPs within the guide RNA has also enabled ASM CRISPRi targeting with a 5–10-fold allelic enrichment in DNAm [308]. Of note, recent experimental evidence has shown that changes introduced to haematopoietic stem cells through experimental CRISPRi manipulation were maintained, propagated, and skewed the lineage development of the descendants of these cells [309].

To date, the experimental in vivo delivery of CRISPRa/i has employed recombinant adeno-associated viral (rAAV) vectors, which benefit from low immunogenicity and high transduction efficiency [310]. However, due to size constraints, the more effective activators have required dual AAV vector approaches to encode the entire module, although these were efficient for local tissue organ-specific effects. Potential improvements could include the investigation of co-packaging of multiple AAVs into extracellular vesicles [311] as well as the use of lentiviral vectors for their increased capacity, although with the trade-off of increased immunogenic activity and reduced barrier crossing and diffusion ability [310]. Notwithstanding these issues, the success so far with these initial forays, hint at the immense future therapeutic potential of these precise epigenomic manipulators.

## Conclusion

### Epigenomic analysis is now integral to pathological diagnostic advances

New insights are forthcoming from combining current diagnostic modalities with multi-omic layers, for example, spatially resolved epigenomics through ATAC profiling in tissue sections, with barcoded solid-phase capture [312]. It is also clear that the current rapid improvements in single molecule long-read sequencing technologies are now starting to deliver on their potential [285], directly analysing DNA as well as RNA modifications [313]. Furthermore, systematic perturbation experiments of CREs, such as enhancers, will substantially improve our functional understanding of the genome [314]. Methods to deal with the sparsity of single cell data will also continue to improve the information acquired at this resolution [315]. Additionally, the power of all this generated ‘big data’ will be utilised by ML and AI for further breakthroughs, with epigenomic data increasingly providing a substantial component for these analyses.

Epigenomic maps of human health and disease are being accumulated, bringing insights to the huge resources now available from robust GWAS findings. However, increased population variation of these epigenomic datasets is needed [181], particularly for understanding the obligatory and facilitative nature of genetic variation’s influence on the epigenome.

Epigenetic biomarkers have the potential to be informative across the breath of clinical applications, for not only cancer, but also other disorders. This includes diagnosis and pathological classification, prognosis, recurrence detection, residual disease, treatment choice and response assessment, population screening, as well as risk stratification [316]. Insights can be gathered from primary tissue biopsies, involving fresh frozen, or Formalin-Fixed Paraffin-Embedded (FFPE) samples, but also derived from cell-free DNA (cfDNA). These ‘liquid biopsy’ cfDNAm analyses can determine the tissue of origin of unknown primary cancers [317], but also assess disease risk, such as pre-eclampsia [318], and specific organ damage, including cardiomyocyte death [319]. Epigenomic biomarkers in surrogate tissues, epidemiologically associated with risk, e.g., tobacco DNAm biomarkers, can also be evaluated, e.g., from blood, sputum, buccal cells, stool, and urine [320]. The plasticity of epigenetic modifications as well as the enriched mutation burden of epigenomic machinery genes has brought a major focus on epidrugs in drug development [316].

In conclusion, epigenomic analysis and diagnostics firstly enable precise molecular understanding of the functional changes of the epigenome in disease. Secondly, using this knowledge can inform the creation of novel sequence-specific regulatory epigenomic therapeutics. Thus, these two facets of epigenomic medicine will lead to substantial improvements in human health.

## Data Availability

Not applicable.
